# Investigation of risk factors associated with impaired glucose regulation: Using the momentum equation to assess the impact of risk factors on community residents

**DOI:** 10.3389/fendo.2023.1145847

**Published:** 2023-03-14

**Authors:** Mengqian Guo, Zhen Wang, Shumei Wang, Jinju Wang, Qiang Jiang

**Affiliations:** ^1^ Department of Traditional Chinese Medicine, Jinan Central Hospital, Jinan, Shandong, China; ^2^ Department of Ophthalmology, Jinan Central Hospital, Jinan, Shandong, China; ^3^ Department of Endocrinology, Jinan Central Hospital, Jinan, Shandong, China

**Keywords:** type 2 diabetes mellitus, impaired glucose regulation, risk factors, general population, hypertension

## Abstract

**Objective:**

To identify risk factors for impaired glucose regulation (IGR) and assess their impact on community residents, this study used a questionnaire to conduct cross-sectional surveys and analysis.

**Methods:**

Overall, 774 residents of an urban community in northern China (Jian city) participated in this study. Trained investigators conducted surveys using questionnaires. Based on their medical history, respondents were divided into three glucose status groups as follows: normal (NGT), IGR, and diabetes mellitus (DM). Statistical analysis of survey data was performed using SPSS v. 22.0.

**Results:**

Age, hypertension, family history of diabetes (FHD), dyslipidemia, obesity, and cardiovascular and cerebral disease (CVD) were positively correlated with IGR in men and women. IGR was negatively correlated with a sedentary lifestyle in men and positively correlated with being overweight in women. The number of type 2 diabetes mellitus (T2D) risk factors per subject was positively correlated with age in the NGT group. Glucose status deteriorated with increasing age and the number of risk factors. FHD was the strongest risk factor in both men and women.

**Conclusions:**

Prevention of IGR includes weight control, physical activity, and prevention of hypertension and dyslipidemia, especially in subjects with FHD.

## 1 Introduction

Type 2 diabetes mellitus (T2D) increases the risk of microvascular and macrovascular complications, and as a result, puts a great economic burden on patients and society. T2D has become an important public health issue worldwide, especially in developing countries (1, 2). Previous studies have indicated that interventions in populations at high risk of developing T2D can effectively prevent diabetes progression (3–8). Unfortunately, despite these efforts, investigations in recent decades have shown that T2D prevalence has gradually increased, as has the prevalence of prediabetes, including impaired glucose tolerance (IGT) and impaired fasting glucose (IFG) (9–13). In China, the incidence of T2D and prediabetes among adults in 2010 was 11.6% and 50.1%, respectively (12). A population-based cross-sectional survey published in 2017 (14) used oral glucose tolerance test (OGTT) data from 43,846 adults aged 20 years or older from the 2007-2008 Chinese Diabetes and Metabolic Disorder Study and resulted in 2,801 newly diagnosed cases of type 2 diabetes. Of these newly diagnosed patients, 654 (23.3%) were aged <43 years and thus had early-onset DM. Early-onset diabetes in China accounts for a higher proportion of new-onset diabetes, suggesting that diabetes in young people has reached pandemic levels. The reasons underlying for this increase are complicated. T2D is the result of interactions between multiple factors including genetic, environmental, lifestyle, nutrition, economic and social factors (15). Most previous studies on T2D prevention have focused on IGT patients, in whom regulation glucose was abnormal at the time of the study (3, 4, 16). Some studies have shown that after a 6-year lifestyle intervention, 73% of people with IGT developed T2D 23 years later (17).

In recent years, IGR has been established as an independent risk factor for acute coronary events (18). Preventive measures from clinical trials cannot eliminate the risk factors or stop the onset and progression of T2D in IGR patients (2, 15). Individuals with normal glucose regulation may also be at risk for T2D (19).

Since IGR is the first stage of T2D, prevention of IGR should be the first stage of T2D prevention, and special attention should be given to risk factors in the general population before glucose regulation in these individuals becomes abnormal. In this study, we conducted a cross-sectional survey to assess the prevalence and ranking of risk factors for IGR and T2D in people with NGR.

There are many risk factors that increase the risk of IGR. Because their prevalence in the community varies, so does their impact on community populations. To evaluate the impact of a risk factor on the population, it is better to consider both the relevance of the risk factor and its incidence in the population. In our study, we used the product of a risk factor’s odds ratio (OR) and its prevalence in the community which we termed “risk momentum” (RM), to assess the impact of each risk factor on the community.

## 2 Methods

### 2.1 Ethical approval

Informed written consent was obtained from all participants prior to enrolment.

### 2.2 Study population

The study population was recruited from an urban residential community (Jian city) in China, from December 2013 to June 2014. The sampling frame consisted of the population living in the community ≥ 2 years and aged ≥15 years. Simple randomization was performed to select participants. A total of 774 subjects participated in the study.

### 2.3 Questionnaire and measurements

The questionnaire on risk factors for T2D (**Table 1**) was designed based on the Guidelines for Prevention and Treatment of Type 2 Diabetes in China (2010 Edition) (20). Trained investigators performed the survey using a published questionnaire (**Table 1**). Subjects were divided into three glucose regulation groups according to their medical history and health records: the diabetic group (the DM group=0, fasting plasma glucose (FPG) ≥7.0 mmol/L and/or 2 hours postprandial blood glucose (P2hBG) ≥11.1 mmol/L, or diagnosed as having T2D by a doctor); the impaired glucose regulation group (the IGR group=1, 6.1 mmol/L≤ FPG <7.0 mmol/L and/or 7.8 mmol/L≤ P2hBG <11.1 mmol/L, or diagnosed as having IGR by a doctor); and the normal group (the NGT group=2, FPG <6.1 mmol/L and P2HBG <7.8 mmol/L). Participants were required to answer the questionnaire based on their latest health reports.

**Table 1:** Questionnaire on risk factors for type 2 diabetes mellitus (T2D).

1. History of impaired glucose regulation (Glucose status) (diabetes = 2, yes = 1, no = 0)
2. Age ≥ 45 years (yes = 1, no = 0)
3. Overweight status and obesity (body mass index (BMI) ≥28 kg/m^2^ was regarded as obese, BMI ≥24 kg/m^2^ was regarded as overweight, and BMI 18–24 kg/m^2^ was regarded as normal) (obesity = 2, overweight = 1, normal = 0)
4. History of T2D in the immediate family (FHD) (yes = 1, no = 0)
5. History of macrosomia (infant birth weight ≥4 kg) (yes = 1, no = 0)
6. History of gestational diabetes mellitus (GDM) (yes = 1, no = 0)
7. History of hypertension (HP, systolic blood pressure ≥140 mmHg and/or diastolic blood pressure ≥ 90 mmHg or current antihypertensive treatment) (yes = 1, no = 0)
8. History of dyslipidemia or current lipid-lowering therapy (yes = 1, no = 0)
9. History of cardiovascular and cerebral disease (CVD) (yes = 1, no = 0);
10. History of glucocorticoid-induced diabetes (GID) (yes = 1, no = 0)
11. History of polycystic ovary syndrome (PCOS) (yes = 1, no = 0)
12. Severe mental disease/long-term use of antidepressants (SMLUA) (yes = 1, no = 0)
13. Sedentary lifestyle, defined as no or very little physical activity during work, housework, transportation, and leisure time. The standard was medium-intensity physical activity of <30 min per day most days of the week (yes = 1, no = 0).

Body mass index (BMI) was calculated by dividing body weight (kg) by the square of height (m). Blood pressure was measured three times in the sitting position with at least 5 minutes of rest before the measurement, and their average was used in the analysis. Past medical history (hypertension, HP; gestational diabetes mellitus, GDM; dyslipidemia; cardiovascular and cerebral disease, CVD; glucocorticoid-induced diabetes, GID; polycystic ovary syndrome, PCOS; and severe mental illness/long-term use of antidepressants, SMLUA) was used based on the previous diagnosis.

### 2.4 Statistical analysis

Statistical analyses were performed using SPSS v. 22.0 (SPSS Inc., Chicago, IL, USA). Continuous variables were summarized and presented as the mean ± standard deviation (SD), and nonparametric variables were presented as numbers and percentages. Only risk factors with an incidence of ≥10% were included in the correlation analysis. Bivariate correlation analyses were performed to identify factors associated with IGR. Factors identified as significant from bivariate analyses were included in a binary logistic regression analysis, and odd ratios with 95% confidence intervals and *p*-values were reported. A bivariate analysis was used to analyze the correlation between the following: the number of T2D risk factors (excluding age) and age (Pearson test); the number of T2D risk factors (excluding age) and glucose status (Spearman test); and glucose status and age (Spearman test). The significance level was set at *p <*0.05, and all analyses were two-sided. The product of a risk factor’s OR value and its prevalence in the community was calculated as RM.

## 3 Results

### 3.1 General information

Of the 774 participants, the NGT group comprised 208 men (aged 47.13 ± 15.61 years) and 381 women (aged 45.92 ± 16.13 years), the IGR group comprised 35 men (aged 60.89 ± 11.44 years) and 48 women (aged 59.27 ± 13.66 years), and the DM group comprised 41 men (aged 62.29 ± 10.08 years) and 61 women (aged 64.03 ± 8.94 years). The prevalence of T2D was 14.4% in men and 12.4% in women. The prevalence of IGR was 12.3% in men and 9.8% in women.

### 3.2 Prevalence of risk factors

The prevalence of T2D risk factors in different groups is shown in **Table 2**. The prevalence of T2D risk factors was higher in the DM and IGR groups than in the NGT group. The prevalence of overweight was the highest among all the factors in both men and women.

**Table 2:** Rank of prevalence of T2D risk factors (on the basis of NGT men group).

T2D Risk Factor	DM		IGR		NGT	
	MAN (%)	WOMAN (%)	MAN (%)	WOMAN (%)	MAN (%)	WOMAN (%)
Overweight	46.3	45.9	48.6	47.9	41.8*†	33.3*†
Sedentary Lifestyle	29.3	39.3	20.0	31.3	33.7	31.5
Hypertension	48.8	75.4	60.0	56.3	24.5*†	20.5*†
FHD	31.7	49.2	48.6	43.8	20.7*	16.3*†
Dyslipidemia	61.0	49.2	51.4	33.3	17.8*†	11.8*†
Obesity	26.8	34.4	22.9	29.2	15.4*†	14.7*†
CVD	39.0	59.0	42.9	47.9	13.5*†	17.3*†
Macrosomia	9.8	21.3	11.4	6.3	7.2	11.3
PCOS	0.0	4.9	0.0	0.0	0.0	0.8
GDM	0.0	1.6	0.0	4.2	0.0	0.3
GID	2.4	1.6	0.0	4.2	0.0	0.5
SMLUA	0.0	4.9	0.0	2.1	0.0	0.3

T2D, type 2 diabetes mellitus; FHD, family history of diabetes; CVD, cardiovascular and cerebral disease; PCOS, polycystic ovary syndrome; GDM, gestational diabetes mellitus; GID, glucocorticoid-induced diabetes; SMLUA, severe mental disease/long-term use of antidepressants; DM, diabetes mellitus group; IGR, impaired glucose regulation group; NGT, normal group.

*Compared with IGR, p <0.05; †compared with DM, p <0.05.

### 3.3 Number of risk factors in each group

Excluding age as a factor, the number of T2D risk factors per person in each group was as follows: DM men, 2.85 ± 1.49 factors and DM women, 3.87 ± 1.52 factors; IGR men, 3.71 ± 1.27 factors and IGR women, 3.81 ± 1.42 factors; NGT men, 1.62 ± 1.37 factors and NGT women, 1.56 ± 1.32 factors. A significant difference in the number of T2D risk factors per person was observed between the NGT group and the other two groups (*p <*0.01), but there was no significant difference between the IGR and DM groups (*p >*0.05). A bivariate Spearman correlation analysis showed that glucose status was negatively correlated with the number of T2D risk factors (excluding age) per person (men, R = −0.435, *p <*0.01; women, R = −0.461, *p <*0.01).

### 3.4 Age

A bivariate Spearman correlation analysis showed that glucose status was negatively correlated with age (R= −0.412, *p <*0.01). The number of T2D risk factors excluding age showed a positive correlation with age in the NGT group using the bivariate Pearson correlation analysis. In the NGT group, Pearson’s correlation coefficient was 0.32 (*p <*0.01) for men and 0.487 (*p <*0.01) for women. In the IGR group, Pearson’s correlation coefficient was −0.128 (*p >*0.05) for men and 0.208 (*p >*0.05) for women. In the DM group, Pearson’s correlation coefficient was 0.249 (*p >*0.05) for men and 0.184 (*p >*0.05) for women.

### 3.5 Risk factors for IGR

All variables with a prevalence of 10% or greater were subjected to bivariate correlation analysis to assess the strength of their association with IGR. Eight variables, including FHD, dyslipidemia, hypertension, CVD, overweight and obesity, age, macrosomia, and sedentary lifestyle, were added to a binary logistic regression model for further analysis (**Table 3**). A sedentary lifestyle showed a negative association with IGR, and overweight was not correlated with IGR in men, in contrast with these variables in women. FHD was the strongest risk factor for IGR in both men and women.

**Table 3:** Binary logistic regression analysis of risk factors for impaired glucose regulation among men and women.

	Men				Women			
Variable	OR	95% of C.I.	*p* value	Product (OR-1)*Prevalence	OR	95% of C.I.	*p* value	Product (OR-1)*Prevalence
Age	1.052	1.047-1.057	0.000		1.043	1.038-1.048	0.000	
Overweight	1.046	0.922-1.186	0.484	1.92	2.140	1.921-2.384	0.000	37.96
Sedentary lifestyle	0.538	0.471-0.614	0.000	-15.57	1.515	1.374-1.670	0.000	16.23
Hypertension	1.848	1.650-2.070	0.000	20.78	1.552	1.406-1.713	0.000	11.32
FHD	3.342	2.982-3.746	0.000	48.48	3.553	3.243-3.893	0.000	41.61
Dyslipidemia	2.949	2.631-3.305	0.000	34.70	1.620	1.473-1.781	0.000	7.32
Obesity	1.374	1.170-1.613	0.000	5.77	2.033	1.803-2.293	0.000	15.19
CVD	1.569	1.388-1.774	0.000	7.68	1.415	1.283-1.561	0.000	7.18
Constant	0.003		0.000		0.004		0.000	

Odds ratio (OR), 95% confidence interval (C.I.) and significance (p value).

FHD, family history of diabetes; CVD, cardiovascular and cerebral disease.

### 3.6 Risk momentum: The product of (OR-1) * the prevalence of risk factors for IGR

**Figure 1:**
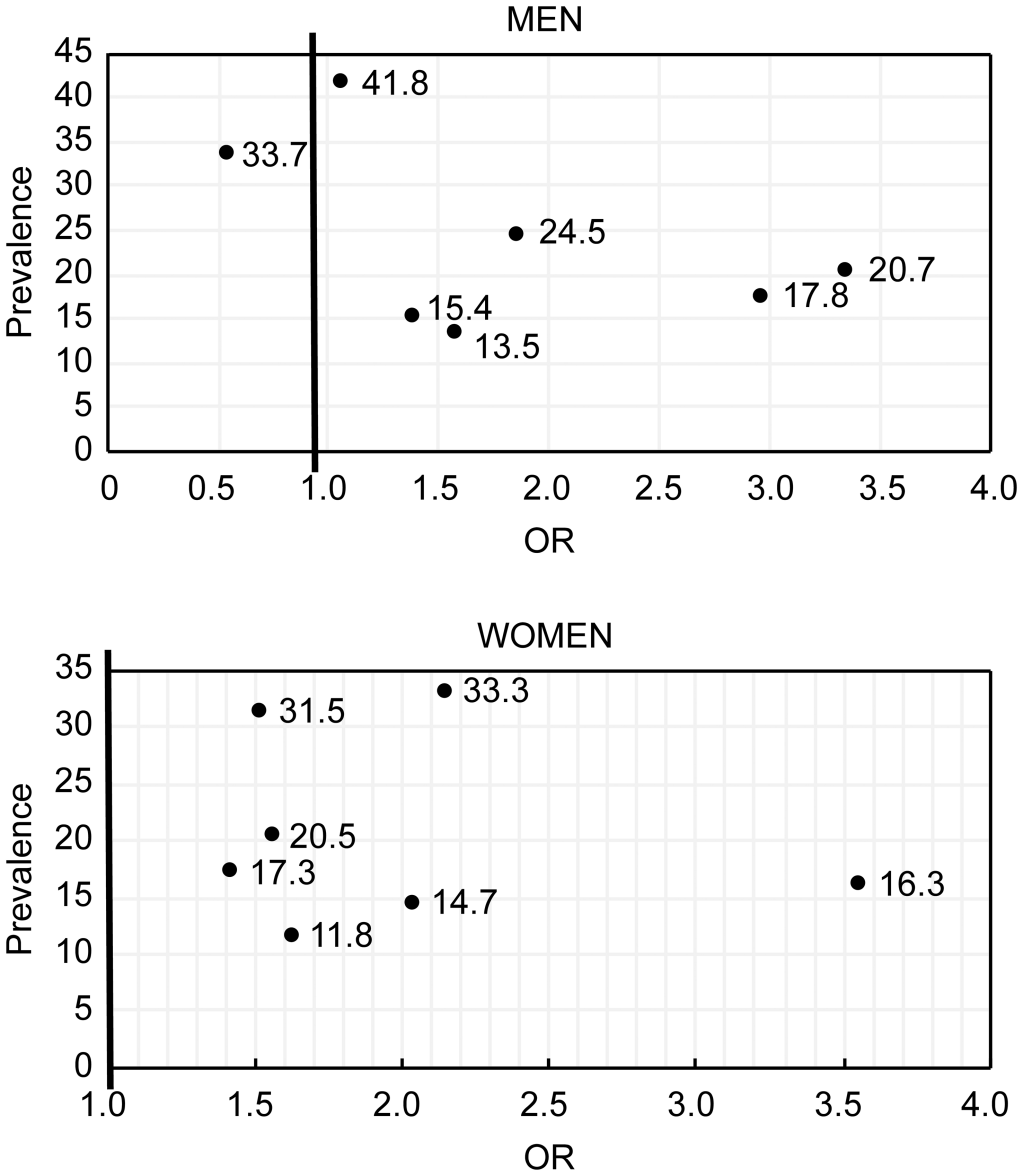
The relationship between the prevalence of risk factors and IGR incidence in men (Ain diagram) and women (top diagram).

In men, FHD had the largest RM, followed by hyperlipidemia and hypertension. In women, FHD also had the largest RM, followed by an overweight, sedentary lifestyle and obesity (**Table 3**; **Figure 1**).

## 4 Discussion

Our survey revealed that age, hypertension, FHD, dyslipidemia, obesity, and CVD were positively correlated with IGR, and a sedentary lifestyle was negatively correlated with IGR in men. In women, age, overweight, sedentary lifestyle, hypertension, FHD, dyslipidemia, obesity, and CVD were positively correlated with IGR. FHD had the strongest correlation with IGR in both men and women. After excluding age as a factor, the number of T2D risk factors was positively correlated with age in the NGT group. Glucose status deteriorated with increasing age and risk factors in the NGT group. FHD had the largest RM in both men and women.

We used IGR prevention as a starting point for T2D prevention. Therefore, we assessed the prevalence of T2D-related risk factors in community residents and identified risk factors associated with IGR to inform IGR prevention strategies. Our data are also consistent with earlier observations suggesting that FHD, obesity, hypertension, dyslipidemia, sedentary lifestyle, and age are correlated with IGR (12, 21).

In our study, FHD had the strongest correlation with IGR both in men and women. The correlation of genetic factors with T2D is known (22, 23). Research has shown that 40% of first-degree relatives of T2D patients develop diabetes, compared with only 6% in the general population (24). Furthermore, monozygotic twins have higher concordance rates (96%) than dizygotic twins in some but not all twin studies (24, 25). The effect of family history may include not only genetic factors but also family aggregation. In addition to genetic factors, family members have similar lifestyles, which could lead to similar T2D risks. The results of a meta-analysis suggest that spousal diabetes can be a diabetc risk factor (26). Although the incidence of FHD was not the highest, the RM of FHD was the largest in both men and women. This finding suggests that FHD might be the most dangerous risk factor for IGR in the community.

We found that being overweight was the most prevalent risk factor in the NGT group. When the prevalence of obesity was considered in addition to the prevalence of overweight, 50% of NGT subjects were overweight. Obesity was significantly correlated with IGR in men and women, but overweight was correlated with IGR only in women. The RM was 37.96 for overweight and 15.19 for obesity in women. This suggests that the criteria for overweight should be defined separately for each gender. Other studies have shown that obesity/overweight plays important role in the progression of DM through adipose tissue factors (27–29). Being overweight (BMI 25-29.9 kg/m2) is significantly correlated with prediabetes (21). A high BMI also correlates with other T2D risk factors (28).

Sedentary lifestyle was the second most prevalent risk factor in the NGT group for both men and women. Other studies have shown sedentary lifestyle to be correlated with diabetes (30). In an investigation on Australian males aged 45 years and older, George ES found that time spent sitting was associated with diabetes, independent of BMI (31). In the present investigation, we found the prevalence of a sedentary lifestyle was not significantly different among the three glucose status groups. Contrary to previous studies (32), we found sedentary lifestyle was positively correlated with IGR in men. This finding might be because doctors generally advise people with IGR to manage their diet and increase physical activity, which could lead to people with IGR performing more physical activity than those without IGR. Moreover, it was well documented that people with IGR can manage the prevalence of T2D *via* diet modification (33). Our findings also indicated that sedentary lifestyles are prevalent throughout the community. The RM of a sedentary lifestyle was the third largest in women. Thus, addressing and reversing this unhealthy lifestyle trend is important.

In this study, the number of T2D risk factors per person was positively correlated with age, and glucose regulation deteriorated with age. Sandovici et al. (34) found changes in gene expression that were linked to T2D in rats. In a 10-year longitudinal study of 10,000 adults in Korea, Ohn et al. (19), found that all participants developed some degree of insulin resistance with age. In the present study, we found that once abnormalities of glucose regulation were present, regardless of IGR or DM, the number of risk factors lost its relevance with age, suggesting that prevention of IGR should begin at a young age.

Dyslipidemia, hypertension and CVD were significantly correlated with IGR in our study. Dyslipidemia had the second largest RM, and hypertension had the third largest RM in men, whereas these factors had the fifth and sixth largest RMs in women. Diabetes and IGR are often accompanied by hypertension, dyslipidemia, and CVD. Hypertension, dyslipidemia and, hyperglycemia are important risk factors for mortality and disease associated with CVD (28, 31, 35). CVD can be considered an indicator of IGR as well as a result of IGR. However, no clear evidence exists as to whether there is a causal relationship between hyperglycemia and dyslipidemia or hypertension, and the underlying connections remain to be determined. Some lifestyle intervention studies have found that T2D incidence decreased with decreasing BMI, blood pressure, and lipid levels (3, 7, 9).

Notably, in our survey, the prevalence of diabetes and prediabetes in the community differed from the latest national survey data (DM, 10.7%; pre-DM, 35.7%) (36). As the incidence of risk factors in different regions may differ, strategies and priorities for the prevention of diabetes and IGR may vary from region to region. Because this was a cross-sectional study, the correlation analysis could not fully account for the long-term effects of risk factors on human physiology. A large longitudinal study is required to validate our findings. In this study, self-reported health information was accepted. Because most Chinese adults conduct general medical examinations only annually, some medical information might have changed between the most recent health examinations and the time when they participated in this study. For instance, patients with latent IGR or T2D might have been missed, which could reduce the effectiveness of our statistical analysis.

## 5 Conclusion

In conclusion, among the analyzed risk factors FHD had the strongest correlation with IGR. Overweight and obesity are the most common risk factors for IGR, and FHD has the largest RM. IGR should be addressed at a young age, especially in those with FHD. The best means of preventing IGR include weight control, prevention of hypertension and dyslipidemia, and consistent physical activity.

## Data availability statement

The raw data supporting the conclusions of this article will be made available by the authors, without undue reservation.

## Ethics statement

The studies involving human participants were reviewed and approved by Medical Ethics Committee of Jinan Central Hospital Affiliated to Shandong University. The patients/participants provided their written informed consent to participate in this study.

## Author contributions

MG: Concept. ZW: Review of data. SW: Correction and revision. JW: Revision of papers. QJ: Conceptualize and writing. All authors contributed to the article and approved the submitted version.
